# Alternative Ultrasound-Assisted Method for the Extraction of the Bioactive Compounds Present in Myrtle (*Myrtus communis* L.)

**DOI:** 10.3390/molecules24050882

**Published:** 2019-03-02

**Authors:** Ana V. González de Peredo, Mercedes Vázquez-Espinosa, Estrella Espada-Bellido, Marta Ferreiro-González, Antonio Amores-Arrocha, Miguel Palma, Gerardo F. Barbero, Ana Jiménez-Cantizano

**Affiliations:** 1 Department of Analytical Chemistry, Faculty of Sciences, University of Cadiz, Agrifood Campus of International Excellence (ceiA3), IVAGRO, 11510 Puerto Real, Cadiz, Spain; ana.velascogope@uca.es (A.V.G.d.P.); mercedes.vazquez@uca.es (M.V.-E.); estrella.espada@uca.es (E.E.-B.); marta.ferreiro@uca.es (M.F.-G.); miguel.palma@uca.es (M.P.); 2 Department of Chemical Engineering and Food Technology, Faculty of Sciences, University of Cadiz, Agrifood Campus of International Excellence (ceiA3), IVAGRO, 11510 Puerto Real, Cadiz, Spain; antonio.amores@uca.es (A.A.-A.); ana.jimenezcantizano@uca.es (A.J.-C.)

**Keywords:** anthocyanins, bioactive compounds, Box–Behnken design, ultrasound-assisted extraction, myrtle, *Myrtus communis* L., phenolic compounds

## Abstract

The bioactive compounds in myrtle berries, such as phenolic compounds and anthocyanins, have shown a potentially positive effect on human health. Efficient extraction methods are to be used to obtain maximum amounts of such beneficial compounds from myrtle. For that reason, this study evaluates the effectiveness of a rapid ultrasound-assisted method (UAE) to extract anthocyanins and phenolic compounds from myrtle berries. The influence of solvent composition, as well as pH, temperature, ultrasound amplitude, cycle and solvent-sample ratio on the total phenolic compounds and anthocyanins content in the extracts obtained were evaluated. The response variables were optimized by means of a Box-Behnken design. It was found that the double interaction of the methanol composition and the cycle, the interaction between methanol composition and temperature, and the interaction between the cycle and solvent-sample ratio were the most influential variables on the extraction of total phenolic compounds (92.8% methanol in water, 0.2 s of cycle, 60 °C and 10:0.5 mL:g). The methanol composition and the interaction between methanol composition and pH were the most influential variables on the extraction of anthocyanins (74.1% methanol in water at pH 7). The methods that have been developed presented high repeatability and intermediate precision (RSD < 5%) and the bioactive compounds show a high recovery with short extraction times. Both methods were used to analyze the composition of the bioactive compounds in myrtle berries collected from different locations in the province of Cadiz (Spain). The results obtained by UAE were compared to those achieved in a previous study where microwave-assisted extraction (MAE) methods were employed. Similar extraction yields were obtained for phenolic compounds and anthocyanins by MAE and UAE under optimal conditions. However, UAE presents the advantage of using milder conditions for the extraction of anthocyanins from myrtle, which makes of this a more suitable method for the extraction of these degradable compounds.

## 1. Introduction

People’s diet is currently improving with a growing demand for healthy food, such as vegetables and fruit. Some berries have been particularly demanded mainly due to their phenolic composition [1,2]. Phenolic compounds are quite prone to oxidation due to their high content in double bonds and hydroxyl groups [3]. This characteristic provides them with a substantial capacity to prevent the oxidation of free radicals, i.e., chemically unstable species that may damage lipid cells, proteins and DNA [4]. Therefore, numerous studies support a positive association between the consumption of berries, which are rich in phenolic compounds, and the prevention against some diseases, such as cardiovascular or neurodegenerative diseases [5]. For that reason, the phenolic compounds extracted from berries are being used by the food, cosmetic and pharmaceutical industries to replace synthetic antioxidants [6,7].

Myrtle (*Myrtus communis* L.), is a widely spread plant throughout the Mediterranean area and the Middle East [8]. This evergreen shrub produces dark blue edible berries of different shapes in most of the characterized ecotypes [9]. These berries are rich in antioxidant compounds, in a considerably greater degree than most other fruit types [10]. Specifically, myrtle berries have a high content of phenolic compounds and anthocyanins [11]. The major phenolic compounds which can be identified in myrtle berries are quercetin 3-*O*-galactoside, quercetin 3-*O*-rhamnoside, myricetin 3-*O*-rhamnoside, quercetin 3-*O*-glucoside, ellagic acid and myricetin [12,13]. The major anthocyanins which can be identified in myrtle berries are delphinidin 3,5-*O*-diglucoside, delphinidin 3-*O*-glucoside, cyanidin 3-*O*-galactoside, cyanidin 3-*O*-glucoside, cyanidin 3-*O*-arabinoside, petunidin 3-*O*-glucoside, delphinidin 3-*O*-arabinoside, peonidin 3-*O*-glucoside, malvidin 3-*O*-glucoside, petunidin 3-*O*-arabinoside and malvidin 3-*O*-arabinoside [14,15]. These compounds, as above mentioned, have exhibited some potentially positive properties for human health, such as antidiabetic, anti-inflammatory, anti-cancer and antioxidant properties [16]. These medicinal properties have meant new fields where myrtle can be used. Consequently, it is currently used in the perfume, cosmetics, healthcare and food industries [17]. In spite of myrtle’s broad potential and the fact that it grows in many extensive areas, its intensive exploitation takes place mainly in Sardinia. In this area, “Mirto” liquor is produced by macerating myrtle leaves and berries [18].

In order to determine the quality control of these beneficial compounds, the development of fast and efficient methods of extraction and analysis is required. Several studies have been carried out on the antioxidant activity and phenolic compounds in the extracts obtained from myrtle berries [19]. Most of these studies use maceration as the extraction technique, while few studies have been found that employ new extraction methods to obtain the extract from myrtle berries [20]. Compared to the commonly used extraction methods, some novel extraction techniques, such as ultrasound-assisted extraction (UAE), microwave-assisted extraction (MAE) or pressurized-liquid extraction (PLE) greatly reduce time, costs and volume of solvent, and also improve the quality of the extracts [21]. In a previous study recently published by the authors, MAE was used for the determination of total phenolic compounds and total anthocyanins in myrtle berries [22]. Based on the results obtained, it was concluded that MAE is an eco-friendlier and easier to use a technique for the extraction of both, phenolic compounds and anthocyanins, from myrtle berries. In this study, UAE is presented as an alternative extraction method, since UAE offers extraction yields comparable to those obtained by means of MAE, but exceeds MAE in terms of the number of solvent used, easiness, and economic cost. The use of ultrasounds is supported by the phenomenon of cavitation. This phenomenon makes of UAE a widely used method, since it breaks cell walls and releases the target compounds out of their natural matrices [23]. For the reason, UAE has been used for the extraction of the antioxidant compounds found in fruits matrices, such as papayas [24], mulberries [25], oranges [26] or sugarcane [27]. With regard to myrtle, some articles have been found in literature where UAE is used to extract biological compounds from myrtle berries [28,29], but it has not yet been carried out a thorough optimization and development of this technique for the specific extraction of phenolic compounds and anthocyanins. This would be very convenience, since the correct applicability would improve the analysis of the raw material, which would enhance the quality, for example, of liquor, as the main product obtained from myrtle. Moreover, the correct applicability of the method would allow to track down the evolution of the fruit’s chemical composition during its maturity process. Reliable maturity indices would be useful to establish optimum harvesting periods, etc.

The aim of the present study is, therefore, to determine optimum conditions for the efficient extraction methods to obtain the greatest possibly yields of substances with antioxidant activities, anthocyanins and phenolic compounds from myrtle berries. Furthermore, the UAE results were compared to those obtained by MAE.

## 2. Results and Discussion

### 2.1. Development of the UAE Method

The Box-Behnken design was applied to the optimization of the variables that mainly influence UAE with regards to total phenolic and total anthocyanins yields. The main variables that affect UAE efficiency are solvent composition, temperature, amplitude of the ultrasound, cycle, pH, and solvent-sample ratio [30]. Table 1 shows such influential variables and their corresponding values studied in this work.

Analysis of variance (ANOVA) was applied to the set of results in order to evaluate the effect of the different factors on their response and the possible interactions between them. Table 2 and Table 3 show the results obtained from this analysis.

**Figure 1:**
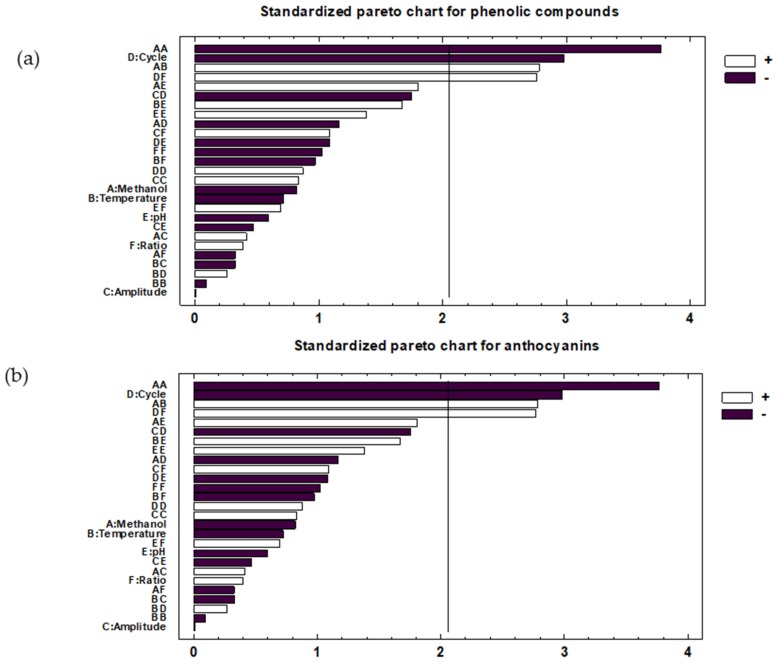
Pareto charts of the standardized effects: (**a**) Total phenolic compounds and (**b**) total anthocyanins.

This information was supplemented with Pareto Charts (Figure 1). Pareto charts show each effect and combination of effects by a bar in decreasing order of significance. From a graphical point of view, this allows to visualize the influencing variables and their degree of influence.

In the case of total phenolic compounds (Figure 1a), cycle (X_4_) was the only linear term which had a significant effect, with a *p*-value lower than 0.01. Its effect on the response variable was negative (b_4_ = −4.17). Numerous studies show that the cycle is an influential variable since the use of ultrasound cycles (pulse) improves the extraction of certain compounds of interest, such as the phenolic compounds in natural matrices [31,32,33]. The negative effect means that a decrease in the cycle increases the extraction of phenolic compounds. This may be due to the negative chemical and physical effects of cavitation [34]. The negative effect is often due to the reactions of free radicals formed during the sonication with molecules in the medium [35], which accelerates the degradation process of phenolic compounds. In addition, the solvent composition had a significant quadratic influence (X_1_^2^) on the response variable (*p*-value < 0.01). The solvent composition is an important variable since it is necessary to extract the phenolic compounds with solvents of similar polarity [36]. Specifically, X_1_^2^ showed a negative effect (b_11_ = −8.06). With regards to interactions between factors, minor interactions between methanol and temperature (X_1_X_2_) (*p*-value < 0.01) and between cycle and solvent-sample ratio (X_4_X_6_) (*p*-value < 0.05) were observed. Both interactions showed positive coefficients (b_12_ = 6.75 and b_46_ = 6.70).

In the case of anthocyanins (Figure 1b), solvent composition (X_1_) was the only linear term that had a significant effect, with a *p*-value lower than 0.01. Its effect on the response variable was positive (b_1_ = 4.80), which indicates that an increase in the methanol percentage in the solvent favored the anthocyanins content in the extract. Many pieces of research have been found in the literature which shows that hydroalcoholic mixtures are more efficient than pure solvents for the extraction of moderately polar molecules, such as phenolic compounds [36]. Phenolic compounds have a moderate polarity, so they are not extracted adequately when pure water mixtures are used (high polarity). The use of methanol increases the solubility of phenolic compounds and the use of water in a lower percentage helps the desorption of the solute from the sample [37]. With regard to the interactions between factors, a minor interaction between methanol and pH (X_1_X_5_) (*p*-value < 0.05) was observed with a positive coefficient (b_15_ = 6.51). These results agree with the bibliographic data [38] where the concentration of organic solvent used and the pH are influential variables on the extraction and stability of anthocyanins from vegetable matrices. Which regard to quadratic effects, non-significant interactions were obtained (*p*-value > 0.05).

The polynomial Equations (1) and (2) for anthocyanins and total phenolic compounds were obtained from the coefficients of the effects and interactions (Table 2 and Table 3). Therefore, two second-order mathematical models were obtained to predict the Y_TA_ and Y_TP_ response values as a function of the independent variables. Lack of fit test showed *p*-values greater than 0.05 for phenolic compounds and for anthocyanins which means that both models fit well.

Y_TA_ (mg·g^−1^) = 23.3422 + 4.80026·X_1_ − 2.04623·X_2_ − 1.88357·X_3_ − 2.47009·X_4_ + 1.13497·X_5_ − 0.111961·X_6_ − 1.95032·X_1_^2^ − 5.59331·X_1_X_2_ − 4.67624·X_1_X_3_ − 2.18294·X_1_X_4_ + 6.50698·X_1_X_5_ + 0.0864269·X_1_X_6_ + 2.22638·X_2_^2^ − 0.145373·X_2_X_3_ − 1.16027·X_2_X_4_ − 3.14005·X_2_X_5_ + 0.811562·X_2_X_6_ + 2.89386·X_3_^2^ + 3.72761·X_3_X_4_ − 1.63114·X_3_X_5_ − 1.04011·X_3_X_6_ − 1.16818·X_4_^2^ − 0.72317·X_4_X_5_ + 0.230451·X_4_X_6_ + 2.02637·X_5_^2^ − 0.956103·X_5_X_6_ − 1.64156·X_6_^2^ (1)

Y_TP_ (mg·g^−1^) = 45.8533 − 1.15407·X_1_ − 1.0103·X_2_ + 0.0166375·X_3_ − 4.17338·X_4_ − 0.837125·X_5_ + 0.549829·X_6_ − 8.06168·X_1_^2^ + 6.75029·X_1_X_2_ + 1.00895·X_1_X_3_ − 1.99896·X_1_X_4_ + 4.38119·X_1_X_5_ −0.796125·X_1_X_6_ − 0.201989·X_2_^2^ − 0.78815·X_2_X_3_ + 0.637275·X_2_X_4_ + 2.87481·X_2_X_5_ − 2.3643·X_2_X_6_ + 1.78604·X_3_^2^ − 4.25675·X_3_X_4_ − 1.14384·X_3_X_5_ + 1.87097·X_3_X_6_ + 1.87383·X_4_^2^ − 2.6411·X_4_X_5_ + 6.70505·X_4_X_6_ + 2.95825·X_5_^2^ + 1.68306·X_5_X_6_ − 2.19536·X_6_^2^ (2)

Both mathematical models can be reduced by omitting the insignificant terms (*p*-value > 0.05). The Equations (3) and (4) of the two reduced models were expressed as follows:

Y_TA_ (mg·g^‒1^) = 23.3422 + 4.80026·X_1_ − 6.60698·X_1_X_5_, (3)

Y_TP_ (mg·g^‒1^) = 45.8533 − 4.17338·X_4_ − 8.06168·X_12_ + 6.75029·X_1_X_2_ + 6.70505·X_4_X_6_ (4)

**Figure 2:**
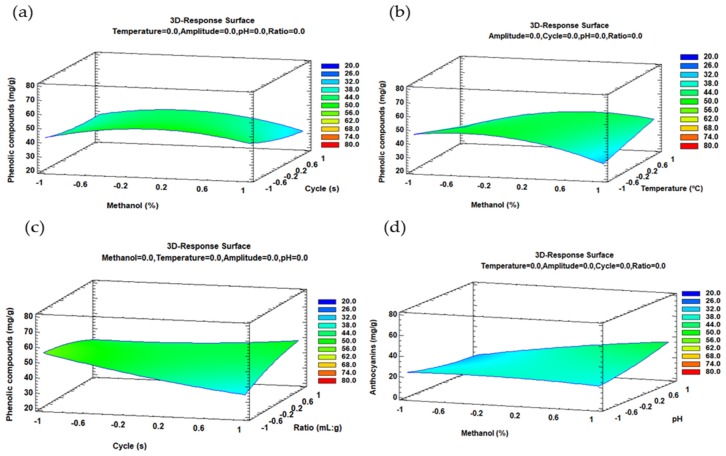
3D-surface plots of the Box–Behnken design to represent the influence of: (**a**) Solvent composition and cycle on the total phenolic compounds; (**b**) solvent composition-temperature on the total phenolic compounds; (**c**) cycle-ratio on the total phenolic compounds; (**d**) solvent composition-pH on the total anthocyanins.

The trends outlined above were recorded in three-dimensional (3D) surface plots using the fitted model in order to improve our understanding of both, the main and the interaction effects, of the most influential parameters. The combined effects of cycle-methanol, methanol–temperature and cycle-ratio on the total phenolic compounds recovery are represented in Figure 2a–c. The combined effect of solvent composition and pH on the total anthocyanins recovery is represented in Figure 2b.

### 2.2. Optimal Conditions

According to the experimental design, the ideal UAE conditions to extract the phenolic compounds were as follows: 92.8% methanol in water as a solvent, 60 °C extraction temperature, 65.48% ultrasound amplitude, 0.2 s cycles, pH 6.8, and 10:0.5 mL:g solvent-sample ratio. With regard to the temperature, no higher temperatures were verified, since they might imply a greater degradation of the compounds of interest and a high loss of methanol that would affect the solvent-sample ratio [39]. With respect to pH, an almost neutral value was determined as optimal, since different research shows that acidified solvents may enhance the formation of free radicals in aqueous solutions because of their higher concentration of H^+^ or thermal treatment [32], which would hinder the recovery of the phenolic compounds [35].

With regards to anthocyanins, optimum UAE conditions were as follows: 74.1% methanol in water solvent, 10 °C extraction temperature, 30% ultrasound amplitude, 0.3 s cycles, pH 7, and 18:0.5 mL:g as the solvent-sample ratio. With respect to temperature, the lowest end of the range studied (10 °C) was determined as the optimum value. Although anthocyanins are also phenolic compounds, they are more thermally sensitive than other phenolic compounds. High temperatures can diminish the recovery of anthocyanins due mainly to oxidation, cleavage of covalent bonds or an increase in oxidation reactions as a result of the thermal treatment [40]. With respect to the solvent pH, neutral pH was found to be optimum for the extraction of anthocyanins. Although pH between 1 and 3 usually generates stable conformation for anthocyanins, there are many articles in the literature where the highest extraction yields take place with a higher pH (3–7) [34]. This behavior might be the effect of different factors on anthocyanins stability (light, temperature, extraction time, etc.), which may turn them into other compounds [41]. Specifically, some authors affirm that ultrasound can promote the degradation of the anthocyanins because of the radical hydroxyl (OH^•^) and *hydrogen peroxide* (H_2_O_2_) produced inside the cavitation bubbles when subjected to conditions, such as high ultrasonic power, high amplitude, low temperature and long treatment time [42]. No higher pH was checked for anthocyanins since this may cause unstable structures as a result of basic hydrolysis [43].

In conclusion, for both, phenolic compounds and anthocyanins, maximum extractions were obtained when the solvent had a high percentage of methanol and neutral pH. Specifically, for the extraction of phenolic compounds a higher range of percentages was required.

### 2.3. Extraction Time

**Figure 3:**
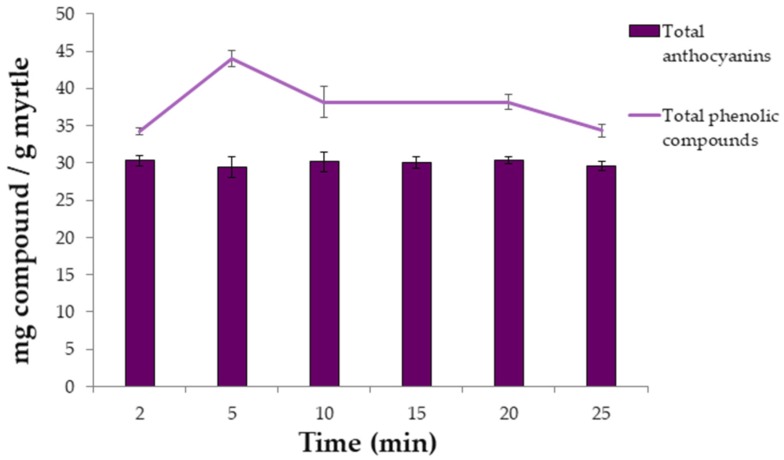
Recovery of anthocyanins (mg·g^−1^) and total phenolic compounds (mg·g^−1^) using different extraction times (*n* = 3).

Once the effects of the variables on the extraction methods and the optimal values were known, the kinetics of the extractions was studied. Several extractions were carried out under optimal ultrasound conditions while extraction time varied between 2, 5, 10, 15, 20, and 25 min. The average results obtained (*n* = 3) for phenolic compounds and for anthocyanins are represented in Figure 3.

It can be seen that large recoveries are achieved for both types of bioactive compounds and that long extraction times are not required. The Phenolic compounds present their maximum extraction at 5 min. However, longer extraction times led to lower recoveries, probably due to degradation of the phenolic compounds [25]. With respect to the anthocyanins, 2 min was determined as their optimum extraction time, since it exhibited the same yields as with longer times, while saving both, time and costs.

### 2.4. Repeatability and Intermediate Precision of UAE Methods

The precision of the extraction methods was evaluated in terms of repeatability (intra-day) and intermediate precision (inter-day). Repeatability was evaluated by performing 10 extractions under the same conditions on the same day. Intermediate precision was evaluated by performing 10 additional extractions on each one of the following two days. Altogether, 30 extractions were carried out under optimal extraction conditions to evaluate the precision of the extraction method for phenolic compounds and for anthocyanins. This method is employed in numerous studies [25,44]. The results were expressed by the coefficient of variation (CV) of the means. The repeatability results obtained were: 2.95% for phenolic compounds and 2.23% for anthocyanins. The intermediate precision results were: 4.66% for phenolic compounds and 4.15% for anthocyanins. As it can be seen, all the results are within acceptable limits (±10%) according to AOAC [45] and supported the accuracy—with diversions lower than 5.0%—of the extraction methods for total anthocyanins and total phenolic compounds.

### 2.5. Application of the Developed Methods to Ecotypes from Two Locations

In order to determine the applicability of the developed methods, once they had been optimized, they were applied to a new set of samples. Specifically, 14 ecotypes of myrtle were evaluated. 8 ecotypes from Puerto Real region (My-1, My-2, My-3, My-4, My-5, My-6, My -7, and My-8) and 6 from San José del Valle region (My-9, My-10, My-11, My-12, My-13, and My-14). The phenolic compounds were extracted from the 14 ecotypes in duplicate by applying the UAE method according to the optimum conditions previously determined. This should ensure the greatest possible yields. The quantification of the total phenolic compounds content in the extracts was carried out by Folin-Ciocalteau reagent.

The anthocyanins compounds were also extracted from the 14 samples according to the optimal conditions determined for the developed UAE method. The anthocyanins content in the extracts was quantified by UHPLC-UV-vis. The total anthocyanins content is the result of adding up each separate anthocyanin content. The average extraction and quantification results are shown in Table 4.

### 2.6. Analysis by Conglomerate

**Figure 4:**
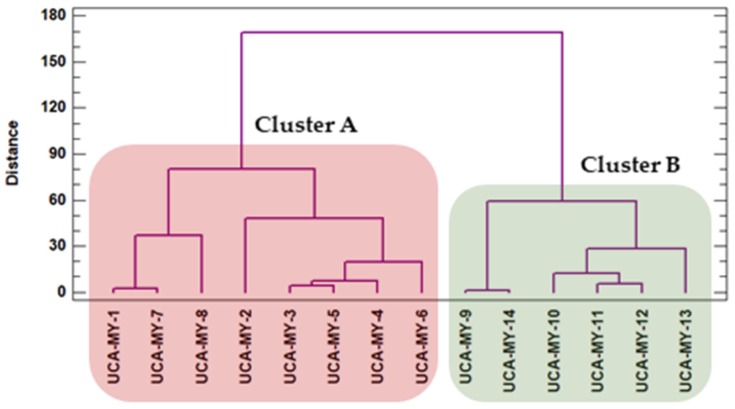
Dendrogram representing the bioactive compounds in 14 myrtle ecotypes according to the matrix of the results from Hierarchical Cluster Analysis (HCA) (Table 3).

As a consequence of the chemical results obtained, it can be observed that there are differences between the average values obtained from the different myrtle ecotypes. To objectively study if these visual differences are related to the origin of the ecotypes, a comparative chemometric study was carried out using all the average values. Specifically, the data matrix D_13 × 14_ (D_variables × ecotypes_) (Table 4) was evaluated using an exploratory tool, i.e., Hierarchical Cluster Analysis (HCA). Ward’s method and square Euclidean distance were employed and the variables for the differentiation were: The total phenolic compounds (mg·g^−1^) from each experiment; each individual anthocyanin content, 11 anthocyanins (mg·g^−1^), and total anthocyanins (mg·g^−1^). Myrtle is a shrub that grows better in warm and humid areas, and requires rich, humid soils. These characteristics match those of Puerto Real, which is near the sea with humid and sandy soils. San José del Valle has drier climate conditions and its clay soil is not so fertile [46,47]. These differences may lead to differences between the maturation processes of the autochthonous ecotypes in Puerto Real and San José del Valle, and consequently, to variations in their bioactive composition. The results of the analysis are graphically represented as a dendrogram in Figure 4. An obvious differentiation of the samples into two groups can be observed: Cluster A, includes only the ecotypes from Puerto Real, and Cluster B, only includes the ecotypes from San José del Valle. Therefore, based on their tendency to fall into a particular group in accordance with their origin, it can be said that phenolic compounds and anthocyanins contents in each ecotype is related to the berries’ geographical area of origin. Specifically, the ecotypes in Cluster A, Puerto Real, present a total phenolic compounds and anthocyanins content greater than the ecotypes in Cluster B, from San José del Valle. The differences can be attributed to the different climatic and soil conditions above mentioned.

### 2.7. Comparison Study: UAE vs. MAE

As above mentioned, the greatest phenolic compounds and anthocyanins yields using UAE were obtained under the following optimal conditions: methanol:water solvent ratio 92.8% *v*/*v* at 60 °C for phenolic compounds and methanol:water solvent ratio 74.1% *v*/*v* at 10 °C for anthocyanins.

In comparison with traditionally used methods for the extraction of the phenolic compounds in myrtle berries, UAE achieves a greater recovery of the compounds of interest, while using less solvent and in a shorter time, with the consequent cost reduction [11,17]. This increased effectiveness with greater extraction yields of both, total phenolic compounds and total anthocyanins, could be based on the phenomenon of cavitation, which breaks cell walls and releases the compounds of interest from the myrtle berries’ matrices.

**Figure 5:**
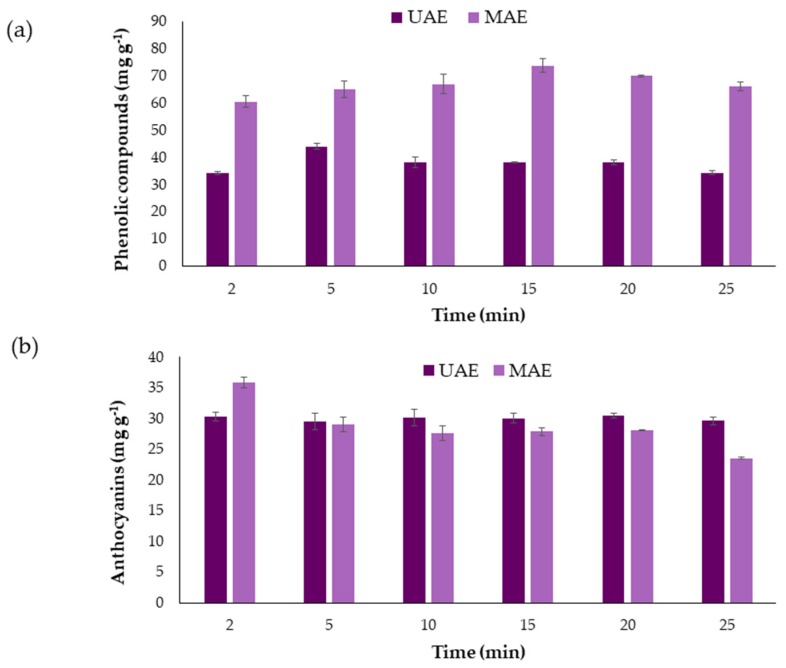
Comparison between the implementation of UAE and microwave-assisted extraction (MAE) to myrtle berries: (**a**) To extract total phenolic compounds; (**b**) to extract anthocyanins.

With the purpose of rounding up this study, the results obtained using UAE were compared to those achieved by MAE in previous work. For that purpose, the same number of samples were run under optimum conditions and later on analyzed [22]. The total phenolic compounds and total anthocyanins content extracted from myrtle berries at different times using UAE and MAE are shown in Figure 5.

With respect to the phenolic compounds (Figure 5a), a similar trend is observed in both extraction methods. The phenolic compounds yield increases until the maximum extraction value is reached at 5 min for UAE and at 15 min for MAE. From then on, the quantity of the extract begins to decrease. The optimum time for UAE, 5 min, indicates that UAE degrades phenolic compounds faster than MAE and the recovery is also lower. When compared to UAE, as recently reported by Ghafoor et al. [48,49], MAE obtains extracts with a substantially greater content of phenolic compounds than the one obtained by UAE.

With respect to the anthocyanins (Figure 5b), their content levels are very similar in MAE and UAE extracts. In addition, the extraction time required to achieve good yields (2 min) is low for both methods, which would considerably reduce costs when operating at an industrial scale. At the optimal time of two minutes, the anthocyanins extraction is slightly higher when MAE is used. However, at longer times, MAE extracts have lower anthocyanins content [50]. MAE optimal operating temperature (50 °C) makes anthocyanins, thermally labile, begin to degrade before.

**Figure 6:**
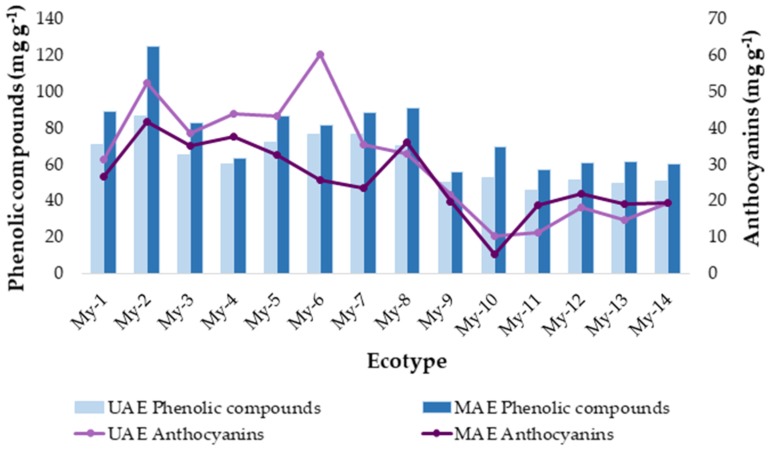
Concentration of total phenolic compounds and total anthocyanins (*n* = 3) in UAE and MAE extracts from several myrtle berries ecotypes.

Additionally, both techniques were applied to different myrtle ecotypes (Figure 6). As above noted, MAE stands out as a more efficient method for the extraction of the phenolic compounds in myrtle berries. With respect to anthocyanins, although some particular ecotypes produced greater yields by MAE, most of them produced greater yields when UAE was employed. Anthocyanins are extremely susceptible to degradation and the combination of high pressure and temperature that is employed for MAE would enhance such degradation and affect negatively their recovery.

Therefore, the UAE should be seriously considered as the preferred method for the extraction of the anthocyanins in myrtle berries.

## 3. Materials and Methods

### 3.1. Plant Materials

The biological materials used for this study were different myrtle berries (14 ecotypes) collected by the authors from two different areas in Cadiz province during their optimum ripeness stages in 2016. The first collection area was Puerto Real (eight ecotypes). This area is characterized by its humid climate due to its proximity to the sea. The second collection area was San José del Valle (6 ecotypes), also within Cadiz province, but located inland at 50 km from the coast. This location has a drier climate and its soils have a lower water content. The guidelines described by M., Mulas and M.R., Cani [47] were applied to characterize the morphology of both, leaves and berries, to confirm that the samples had been collected from different ecotypes. The samples were subjected to a pretreatment to improve the contact surface with the solvent [51]. First, the seeds were separated from the pulp. Secondly, the pulp was lyophilized in a Virtis Benchtop K freeze dryer (SP Cientific, New York, United States) and crushed by means of a regular spice grinder. Finally, the samples were stored in a freezer at −20 °C prior to analysis.

### 3.2. Chemicals and Solvents

The solvents used for the extraction were a mix of methanol and water at different concentration levels and with different pH. The methanol (Fischer Scientifics, Loughborough, United Kingdom) was HPLC grade. Ultra-pure water was obtained from a Milli-Q water purification system (EMD Millipore Corporation, Bedford, MA, United States). The pH adjustment of the solvents was done by means of hydrochloric acid and sodium hydroxide, both analytical grade and purchased from Panreac Química S.A.U. (Castellar del Valles, Barcelona, Spain). For the Folin-Ciocalteau spectrophotometric method, anhydrous sodium carbonate (Panreac Química S.A.U., Castellar del Valles) and Folin–Ciocalteu (Merck KGaA, EMD Millipore Corporation, Darmstadt, Germany) were employed. For the HPLC analyses, methanol (Fischer Scientific, Loughborough, United Kingdom) and formic acid (Scharlau, Barcelona, Spain) were used. These solvents were degassed and filtered through a 0.22 µm membrane (Nylon Membrane Filter, FILTER-LAB, Barcelona, Spain) before being used. The standard for the phenolic compounds was gallic acid and the standard for anthocyanins was cyanidin chloride. Both standards were purchased from Sigma-Aldrich Chemical Co. (St. Louis, MO, USA).

### 3.3. Ultrasound-Assisted Extraction Procedure

To extract the total phenolic compounds and the total anthocyanins from the myrtle berries, UAE was used. A UP200S probe (Hielscher Ultrasound Technology, Berlin, Germany) was employed, coupled to a processor that allows adjusting the amplitude and the cycle. For the adjustment of the temperature, a thermostatic bath (Frigiterm-10, Selecta, Barcelona, Spain) was employed. The temperature, the cycle, and the amplitude were selected for each extraction according to the experiment. About 0.5 g of the lyophilized and homogenized sample was weighed in a Falcon tube and the corresponding volume of solvent was added depending on the experiment. The Falcon tube was placed in a double vessel through which the water from the thermostatic bath circulated. The initial extraction time set was 10 min, followed by a sample cooling time. After that time, the extracts were centrifuged (7500 rpm, 5 min) and the supernatants were placed in a 25 mL volumetric flask. The precipitates from the extraction were redissolved in 5 mL of the same extraction solvent and centrifuged again under the same conditions. The new supernatants were placed in the volumetric flask and it was completed with the same solvent. The final extracts were stored at −20 °C for their correct conservation until further analysis. The UAE conditions set for the extractions were: Solvent composition (50–100% methanol in water for phenolic compounds and 25–75% for anthocyanins), temperature (10–60 °C), amplitude (30–70%), cycle (0.2–0.7 s), pH (2–7) and solvent-sample ratio (10:0.5–20:0.5 mL:g).

### 3.4. Determining the Content of Total Phenolic Compounds by Folin-Ciocalteau Essay

The total phenolic compounds content in myrtle berry was determined by adapted/modified Folin-Ciocalteau (FC) method [52]. This method has been previously used by many researchers to determine the total phenolic compounds content [53,54]. It is based on a redox reaction in a basic medium that gives rise to a complex of blue coloration with a wide absorption up to 765 nm. The extracts were filtered using a 0.45 µm nylon syringe filter (Membrane Solutions, Dallas, United States). The protocol of the method is the following: 250 µL of the previously filtered extract was transferred to a 25 mL volumetric flask. After this, 12.5 mL of water, 1.25 mL of Folin-Ciocalteau reagent and 5 mL of a 20% aqueous sodium carbonate solution were added. Finally, the flask was made up with water, and after 30 min the absorbance was measured at the maximum. All the extracts were analyzed in duplicate. The range of absorbance obtained for the studied samples was 0.4-1.4. The equipment used to measure the absorbance was a Helios Gamma (γ) Unicam UV-vis Spectrophotometer (Thermo Fisher Scientific, Waltham, MA, United States). The calibrated curve for the quantification was constructed based on a reference standard gallic acid pattern under the same conditions as the extracts [55]. The computer application used to process the data was Microsoft Office Excel 2013. The following regression equation y = 0.0010x + 0.0059 and the following correlation coefficient R^2^ = 0.9999 were obtained. The linear range of work was 100–2600 mg L^−1^. The results are expressed in milligrams of gallic acid equivalent per gram of lyophilized weight.

### 3.5. Identification of Anthocyanins by UHPLC-QToF-MS

**Figure 7:**
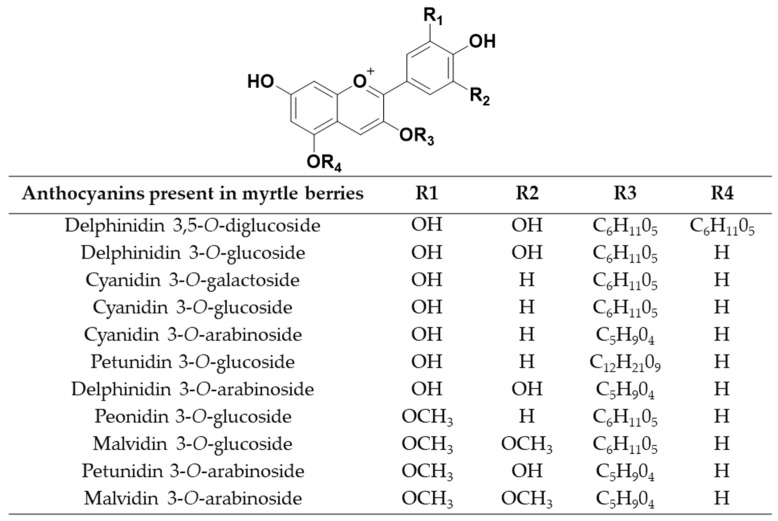
Radicals of the different anthocyanins present in myrtle berries.

Ultra-high performance liquid chromatography (UHPLC) coupled to quadrupole-time-of-flight mass spectrometry (QToF-MS) (Xevo G2 QToF, Waters Corp., Milford, MA, United States) was used to identify the anthocyanins in the UAE extracts. The column employed was a reverse-phase C18 analytical column with 1.7 μm particle size, 2.1 mm × 100 mm (ACQUITY UPLC CSH C18, Waters). The mobile phase was 2% formic acid–water solution (phase A) and methanol solution (phase B). The studied bioactive compounds were determined by employing the UHPLC-QToF-MS method described in a previously research [22]. The individual anthocyanins were identified based on their retention time and molecular weight. The following eleven anthocyanins were identified in the samples: delphinidin 3,5-*O*-diglucoside (*m*/*z* = 627.1561), delphinidin 3-*O*-glucoside (*m/z* = 465.1033), cyanidin 3-*O*-galactoside (*m*/*z* = 449.1084), cyanidin 3-*O*-glucoside (*m*/*z* = 449.1084), cyanidin 3-*O*-arabinoside (*m*/*z* = 419.0978), petunidin 3-*O*-glucoside (*m*/*z* = 479.1189), delphinidin 3-*O*-arabinoside (*m*/*z* = 435.0927), peonidin 3-*O*-glucoside (*m*/*z* = 463.1240), malvidin 3-*O*-glucoside (*m*/*z* = 493.1346), petunidin 3-*O*-arabinoside (*m/z* = 449.1084) and malvidin 3-*O*-arabinoside (*m*/*z* = 463.1240). Before their identification, all the UAE extracts were filtered through a 0.20 μm nylon syringe filter (Membrane Solutions, Dallas, TX, United States). The anthocyanins structures are shown in Figure 7.

### 3.6. Determination of Anthocyanins by UHPLC-UV-Vis System

**Figure 8:**
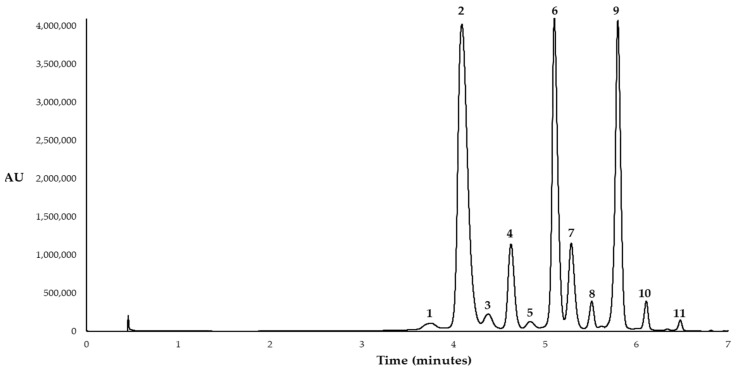
Chromatogram of the 11 anthocyanins identified in the UAE extracts from myrtle berries. Peak assignment: (1) Delphinidin 3,5-*O*-diglucoside; (2) delphinidin 3-*O*-glucoside; (3) cyanidin 3-*O*-galactoside; (4) cyanidin 3-*O*-glucoside; (5) cyanidin 3-*O*-arabinoside; (6) petunidin 3-*O*-glucoside; (7) delphinidin 3-*O*-arabinoside; (8) peonidin 3-*O*-glucoside; (9) malvidin 3-*O*-glucoside; (10) petunidin 3-*O*-arabinoside; (11) malvidin 3-*O*-arabinoside.

For the separation and quantification of the anthocyanins present in UAE extracts from myrtle berries, an Elite UHPLC LaChrom Ultra System (Hitachi, Tokyo, Japan) was used. The UHPLC system consists of an L-2420U UV-Vis detector, an L-2200U autosampler, an L-2300 column oven set at 50 °C and two L-2160 U pumps. The column used was a “Fused Core” C18 with 2.6 μm particle size, 2.1 mm × 100 mm (Phenomenex Kinetex, Torrance, CA, United States). The mobile phase consisted of a 5% formic acid–water solution (phase A) and a methanol solution (phase B). The studied bioactive compounds were determined by employing the UHPLC-UV-Vis method described in previous research [22]. Before their analysis, all the UAE extracts were filtered through a 0.20 µm nylon syringe filter (Membrane Solutions, Dallas, TX, United States) and diluted in Milli-Q water. The individual anthocyanins present in myrtle extracts were quantified in cyanidin equivalents by means of a regression curve of anthocyanidin standard, cyanidin chloride (y = 252640.4136x − 28462.4337; R^2^ = 0.9999). The standards with a known concentration were prepared between 0.06 and 35 mg·L^−1^. The limit of detection (LOD) (0.196 mg·L^−1^) and the limit of quantification (LOQ) (0.653 mg·L^−1^) were calculated as three and ten times respective to the standard deviation of the blank divided by the slope of the calibration curve. Assuming that the 11 anthocyanins have similar absorbance, and taking into account the molecular weight of each anthocyanin, a calibration curve was plotted for each anthocyanin present in myrtle, which allowed to quantify the compounds of interest. All the analyses were carried out in duplicate. Figure 8 shows the HPLC chromatogram that represents the eleven anthocyanins detected in the analyses.

### 3.7. Application of Box-Behnken Design (BBD) to the Optimization of the Extraction Methods

In order to optimize the extraction variables, a response surface experiment (RSM) known as Box-Behnken (BBD) was carried out [56]. Box-Behnken design (BBD) is an independent rotatable quadratic design with no embedded factorial or fractional factorial points. The variable combinations are at the midpoint of the edges and at the center of the space [57]. It is useful because it allows one to avoid carrying out experiments under extreme conditions and, therefore, the possibility of deceiving results [58]. When this statistical experiment design is employed in conjunction with a response surface methodology (RSM) the effects of six independent factors on each response can be studied. The independent factors studied were: Solvent composition (% methanol in water) (X_1_), solvent pH (X_2_), extraction temperature (X_3_), ultrasound amplitude (X_4_), cycle (X_5_), and a solvent-sample ratio (X_6_). For each variable, there are three levels, coded as –1 (low), 0 (central point or middle), and +1 (high). Specifically, the studied ranges were as follows: Solvent composition: 50, 75, 100% for phenolic compounds and 25, 50, 75% for anthocyanins; temperature: 10, 35, 60 °C; amplitude: 30, 50, 70%; cycle: 0.2, 0.45, 0.7 s; pH: 2, 4.5, 7 and solvent-sample ratio: 10:0.5, 15:0.5, 20:0.5 mL:g. The ranges for the study were selected taking into account previous experiences by the research team. The response variables studied were: The experimental results for total phenolic compounds (Y_TP_, mg·g^−1^) and the experimental results for total anthocyanins (Y_TA_, mg·g^−1^). The design consisted of 54 treatments with six repetitions at the center point. All the trials were performed in random order. The whole experimental design matrix used can be seen in Table 5. My-9 from San Jose del Valle was the myrtle sample used for the optimization procedure.

The results for total phenolic compounds and total anthocyanins contents were entered into a polynomial equation. The response of the total phenolic compounds and the anthocyanins obtained in each of the experiments was entered into a second-order polynomial equation in order to correlate the relationship between the independent variables and the response (Equation (5)):

(5) $$Y=\;\beta_{0}+\overset{k}{\underset{i=1}{\sum }}\beta_{i}X_{i}+\;\beta_{ii\;}X_{i}^{2}+\;\underset{i}{\sum }\overset{k}{\underset{i=1}{\sum }}\beta_{ij}X_{i}X_{j}+r$$

where Y is the predicted response (Y_TP_ and Y_TA_); β_0_ is the model constant; X_i_ and X_j_ are the independent variables; β_i_ are the linear coefficients; β_ij_ are the coefficients corresponding to the interactions; βii are the quadratic coefficients and r is the pure error sum of squares.

Design Expert software 11 (Trial Version, Stat-Ease Inc., Minneapolis, MN, USA) was the software employed for experimental design, the data analysis, and the model building. The statistical significance of the model, lack of fit, and regression terms were evaluated based on the analysis of variance (ANOVA).

The results of applying the extraction method to different myrtle ecotypes were studied using a multivariate analysis, hierarchical clustering analysis (HCA). Ward’s method and the Euclidean square distance, were employed. Statgraphic Centurion XVII (Statgraphics Technologies, Inc., The Plains, VA, United States) was the software used.

## 4. Conclusions

This work has successfully developed quick and effective methods to extract bioactive compounds, such as anthocyanins and total phenolics from myrtle (Myrtus communis L.) pulp. A thorough search in the relevant literature showed that this is the first study in which UAE has been optimized for the extraction of phenolic compounds and anthocyanins from myrtle berries. The following optimal UAE conditions have been determined to extract the phenolic compounds: 92.8% methanol in water, 6.8 pH, 60 °C temperature, 65.48% ultrasound amplitude, 0.2 s cycle, and 10:0.5 as the optimum solvent-sample ratio. With regards to anthocyanins, optimal UAE conditions were: 74.1% methanol in water, 7 pH, 10 °C temperature, 30% ultrasound amplitude, 0.3 s cycle, and 18:0.5 as the optimum solvent-sample ratio. The optimum extraction times were only 5 and 2 min for phenolic compounds and anthocyanins, respectively. Both extraction methods presented satisfactory intra-day repeatability and inter-day repeatability (CV < 5%). The methods were applied to 14 different myrtle ecotypes. The hierarchical cluster analysis (HCA), showed a correlation between the bioactive composition (total phenolic compounds and total and individual anthocyanins contents in the extracts) and the ecotypes’ geographical area of origin. In conclusion, the results have indicated that UAE is a feasible alternative to conventional methods for the extraction of valuable components from myrtle berries. These results would mean a substantial improvement at the industrial level, since they would allow the manufacturers to quickly determine the quality of the raw materials and save costs. Furthermore, UAE results were compared to those achieved by MAE. The proposed UAE method proved to be an effective procedure to extract the bioactive compounds in myrtle berries, and a particularly efficient alternative for the extraction of anthocyanins.

## Figures and Tables

**Table 1:** Influential variables with their corresponding values studied in this work.

Variables	Studied Ranges	
	Phenolic Compounds	Anthocyanins
Temperature (°C)	10, 35, 60	10, 35, 60
Amplitude (%)	30, 50, 70	30, 50, 70
Cycle (s)	0.2, 0.45, 0.7	0.2, 0.45, 0.7
pH	2, 4.5, 7	2, 4.5, 7
Solvent-sample ratio (mL/0.5 g)	10, 15, 20	10, 15, 20
Solvent composition (% methanol in water)	50, 75, 100	25, 50, 75

**Table 2:** ANOVA for the quadratic model adjusted to the extraction of total phenolic compounds.

	Source	Coefficient	Sum of Squares	Degrees of Freedom	Mean Square	*F*-Value	*p*-Value
	Model		2940.04	27	108.89	2.31	0.0179
Methanol	X_1_	−1.15	31.97	1	31.97	0.6787	0.4175
Temperature	X_2_	−1.01	24.50	1	24.50	0.5202	0.4772
Amplitude	X_3_	0.0166	0.0066	1	0.0066	0.0001	0.9906
Cycle	X_4_	−4.17	418.01	1	418.01	8.88	0.0062
pH	X_5_	−0.8371	16.82	1	16.82	0.3571	0.5553
Ratio	X_6_	0.5498	7.26	1	7.26	0.1541	0.6979
Methanol × Temperature	X_1_X_2_	6.75	364.53	1	364.53	7.74	0.0099
Methanol × Amplitude	X_1_X_3_	1.01	8.14	1	8.14	0.1729	0.6809
Methanol × Cycle	X_1_X_4_	−2.00	63.93	1	63.93	1.36	0.2545
Methanol × pH	X_1_X_5_	4.38	153.56	1	153.56	3.26	0.0826
Methanol × Ratio	X_1_X_6_	−0.7961	5.07	1	5.07	0.1077	0.7454
Temperature × Amplitude	X_2_X_3_	−0.7882	4.97	1	4.97	0.1055	0.7479
Temperature × Cycle	X_2_X_4_	0.6373	3.25	1	3.25	0.0690	0.7949
Temperature × pH	X_2_X_5_	2.87	132.23	1	132.23	2.81	0.1058
Temperature × Ratio	X_2_X_6_	−2.36	44.72	1	44.72	0.9495	0.3388
Amplitude × Cycle	X_3_X_4_	−4.26	144.96	1	144.96	3.08	0.0911
Amplitude × pH	X_3_X_5_	−1.14	10.47	1	10.47	0.2222	0.6413
Amplitude × Ratio	X_3_X_6_	1.87	56.01	1	56.01	1.19	0.2855
Cycle × pH	X_4_X_5_	−2.64	55.80	1	55.80	1.18	0.2863
Cycle × Ratio	X_4_X_6_	6.71	359.66	1	359.66	7.64	0.0104
pH × Ratio	X_5_X_6_	1.68	22.66	1	22.66	0.4812	0.4940
Methanol × Methanol	X_1_^2^	−8.06	668.48	1	668.48	14.19	0.0009
Temperature × Temperature	X_2_^2^	−0.2020	0.4197	1	0.4197	0.0089	0.9255
Amplitude × Amplitude	X_3_^2^	1.79	32.81	1	32.81	0.6967	0.4115
Cycle × Cycle	X_4_^2^	1.87	36.12	1	36.12	0.7669	0.3892
pH × pH	X_5_^2^	2.96	90.01	1	90.01	1.91	0.1786
Ratio × Ratio	X_6_^2^	−2.20	49.57	1	49.57	1.05	0.3144
Residual		45.85	1224.48	26	47.10		
Lack of Fit			1075.70	21	51.22	1.72	0.2858
Pure Error			148.78	5	29.76		
Total			4164.52	53			

**Table 3:** ANOVA for the quadratic model adjusted to the extraction of total anthocyanins.

	Source	Coefficient	Sum of Squares	Degrees of Squares	Mean Square	*F*-Value	*p*-Value
	Model		2514.45	27	93.13	1.47	0.1631
Methanol	X_1_	4.80	553.02	1	553.02	8.75	0.0065
Temperature	X_2_	−2.05	100.49	1	100.49	1.59	0.2185
Amplitude	X_3_	−1.88	85.15	1	85.15	1.35	0.2563
Cycle	X_4_	−2.47	146.43	1	146.43	2.32	0.1400
pH	X_5_	1.13	30.92	1	30.92	0.4892	0.4905
Ratio	X_6_	−0.1120	0.3008	1	0.3008	0.0048	0.9455
Methanol × Temperature	X_1_X_2_	−5.59	250.28	1	250.28	3.96	0.0572
Methanol × Amplitude	X_1_X_3_	−4.68	174.94	1	174.94	2.77	0.1082
Methanol × Cycle	X_1_X_4_	−2.18	76.24	1	76.24	1.21	0.2821
Methanol × pH	X_1_X_5_	6.51	338.73	1	338.73	5.36	0.0288
Methanol × Ratio	X_1_X_6_	0.0864	0.0598	1	0.0598	0.0009	0.9757
Temperature × Amplitude	X_2_X_3_	−0.1454	0.1691	1	0.1691	0.0027	0.9591
Temperature × Cycle	X_2_X_4_	−1.16	10.77	1	10.77	0.1704	0.6831
Temperature × pH	X_2_X_5_	−3.14	157.76	1	157.76	2.50	0.1262
Temperature × Ratio	X_2_X_6_	0.8116	5.27	1	5.27	0.0834	0.7751
Amplitude × Cycle	X_3_X_4_	3.73	111.16	1	111.16	1.76	0.1963
Amplitude × pH	X_3_X_5_	−1.63	21.28	1	21.28	0.3368	0.5667
Amplitude × Ratio	X_3_X_6_	−1.04	17.31	1	17.31	0.2739	0.6052
Cycle × pH	X_4_X_5_	−0.7232	4.18	1	4.18	0.0662	0.7990
Cycle × Ratio	X_4_X_6_	0.2305	0.4249	1	0.4249	0.0067	0.9353
pH × Ratio	X_5_X_6_	−0.9561	7.31	1	7.31	0.1157	0.7365
Methanol × Methanol	X_1_^2^	−1.95	39.12	1	39.12	0.6191	0.4385
Temperature × Temperature	X_2_^2^	2.23	50.98	1	50.98	0.8068	0.3773
Amplitude × Amplitude	X_3_^2^	2.89	86.14	1	86.14	1.36	0.2536
Cycle × Cycle	X_4_^2^	−1.17	14.04	1	14.04	0.2221	0.6414
pH × pH	X_5_^2^	2.03	42.24	1	42.24	0.6683	0.4211
Ratio × Ratio	X_6_^2^	−1.64	27.72	1	27.72	0.4386	0.5136
Residual		23.34	1643.02	26	63.19		
Lack of Fit			1469.28	21	69.97	2.01	0.2248
Pure Error			173.75	5	34.75		
Total			4157.47	53			

**Table 4:** Extract concentrations (mg·g^−1^) of total anthocyanins and total phenolic compounds (*n* = 3) obtained from different myrtle ecotypes by means of ultrasound-assisted method (UAE).

Compounds ^1^	Myrtle ecotypes from Puerto Real								Myrtle ecotypes from San José del Valle					
	MY-1	MY-2	MY-3	MY-4	MY-5	MY-6	MY-7	MY-8	MY-9	MY-10	MY-11	MY-12	MY-13	MY-14
**D-3,5-diGl**	0.166 ± 0.006	0.415 ± 0.016	0.404 ± 0.015	0.504 ± 0.020	0.377 ± 0.014	0.375 ± 0.016	0.183 ± 0.008	0.103 ± 0.0034	0.127 ± 0.004	0.065 ± 0.002	0.079 ± 0.0023	0.176 ± 0.0067	0.113 ± 0.003	0.134 ± 0.004
**Del-3-Glu**	10.552 ± 0.161	16.309 ± 0.534	10.305 ± 0.358	11.114 ± 0.384	12.557 ± 0.481	12.258 ± 0.432	8.315 ± 0.326	5.929 ± 0.165	3.667 ± 0.152	1.566 ± 0.008	2.145 ± 0.084	3.823 ± 0.124	2.987 ± 0.121	3.346 ± 0.135
**Cy-3-Ga**	0.150 ± 0.006	0.382 ± 0.008	0.216 ± 0.009	0.359 ± 0.007	0.319 ± 0.012	0.371 ± 0.0135	0.176 ± 0.007	0.392 ± 0.012	0.050 ± 0.001	0.052 ± 0.002	0.068 ± 0.0018	0.1757 ± 0.005	0.077 ± 0.002	0.036 ± 0.001
**Cy-3-Gl**	1.794 ± 0.084	3.004 ± 0.15	1.126 ± 0.023	1.241 ± 0.036	1.613 ± 0.03	1.313 ± 0.0578	1.946 ± 0.034	0.791 ± 0.043	0.852 ± 0.024	0.293 ± 0.01	0.301 ± 0.016	0.512 ± 0.025	0.452 ± 0.013	0.786 ± 0.032
**Cy-3-Ar**	0.092 ± 0.003	0.126 ± 0.002	0.076 ± 0.002	0.130 ± 0.002	0.112 ± 0.004	0.170 ± 0.006	0.061 ± 0.14	0.081 ± 0.0025	0.170 ± 0.003	0.040 ± 0.001	0.410 ± 0.021	0.657 ± 0.027	0.549 ± 0.23	0.188 ± 0.006
**Pet-3-Gl**	6.217 ± 0.110	11.007 ± 0.38	7.173 ± 0.314	8.691 ± 0.301	8.329 ± 0.297	10.242 ± 0.392	5.538 ± 0.0423	5.082 ± 0.176	6.810 ± 0.235	1.580 ± 0.018	1.676 ± 0.065	2.123 ± 0.085	1.823 ± 0.067	6.643 ± 0.114
**Del-3-Ara**	0.840 ± 0.005	2.378 ± 0.102	1.635 ± 0.062	1.775 ± 0.067	1.917 ± 0.064	2.009 ± 0.081	1.335 ± 0.021	1.562 ± 0.035	1.833 ± 0.076	0.367 ± 0.006	0.324 ± 0.009	0.679 ± 0.021	0.454 ± 0.014	1.234 ± 0.033
**Peo-3-Gl**	1.030 ± 0.017	1.179 ± 0.043	0.659 ± 0.019	0.689 ± 0.021	0.610 ± 0.023	0.816 ± 0.029	0.755 ± 0.34	0.481 ± 0.019	0.473 ± 0.015	0.305 ± 0.017	0.357 ± 0.012	0.921 ± 0.032	0.564 ± 0.013	0.446 ± 0.012
**Mal-3-Gl**	9.834 ± 0.314	16.691 ± 0.641	16.050 ± 0.65	18.301 ± 0.614	12.748 ± 0.768	23.817 ± 1.032	8.244 ± 0.273	9.072 ± 0.348	6.987 ± 0.246	5.195 ± 0.068	5.453 ± 0.185	7.679 ± 0.23	6.456 ± 0.241	5.979 ± 0.263
**Pet-3-Ar**	0.269 ± 0.009	0.551 ± 0.034	0.448 ± 0.014	0.587 ± 0.013	0.482 ± 0.028	0.751 ± 0.0013	0.332 ± 0.012	0.583 ± 0.015	0.438 ± 0.017	0.197 ± 0.002	0.206 ± 0.008	0.657 ± 0.023	0.454 ± 0.012	0.446 ± 0.012
**Mal-3-Ar**	0.226 ± 0.004	0.302 ± 0.009	0.402 ± 0.012	0.436 ± 0.121	0.296 ± 0.015	0.598 ± 0.019	0.240 ± 0.009	0.395 ± 0.014	0.185 ± 0.005	0.221 ± 0.001	0.244 ± 0.012	0.846 ± 0.032	0.679 ± 0.031	0.165 ± 0.006
**Total anthocyanins**	31.170 ± 1.023	52.346 ± 1.124	38.493 ± 3.516	43.827 ± 2.557	43.274 ± 1.904	60.252 ± 0.002	35.207 ± 1.175	32.903 ± 1.162	21.591 ± 0.92	10.381 ± 0.037	11.263 ± 0.431	18.249 ± 2.30	14.608 ± 1.964	19.403 ± 1.97
**Total Phenolic compounds**	70.747 ± 1.433	86.439 ± 3.125	64.7586 ± 0.914	60.034 ± 0.44	72.155 ± 3.174	76.124 ± 1.170	76.070 ± 3.417	69.815 ± 2.947	49.824 ± 0.362	52.66 ± 0.038	45.789 ± 1.824	51.278 ± 2.051	49.103 ± 0.192	50.897 ± 1.564

^1^ Del-3,5-diGl, delphinidin 3,5-*O*-diglucoside; Del-3-Glu, delphinidin 3-*O*-glucoside; Cy-3-Ga, cyanidin 3-*O*-galactoside; Cy-3-Gl, cyanidin 3-*O*-glucoside; Cy-3-Ar, cyanidin 3-*O*-arabinoside; Pet-3-Gl, petunidin 3-*O*-glucoside; Del-3-Ara, delphinidin 3-*O*-arabinoside; Peo-3-Gl, peonidin 3-*O-*glucoside; Mal-3-Gl, malvidin 3-*O*-glucoside; Pet-3-Ar, petunidin 3-*O*-arabinoside; Mal-3-Ar, malvidin 3-*O*-arabinoside.

**Table 5:** Experimental and predicted values for total phenolic compounds and total anthocyanins contents based on Box–Behnken design.

Run	Factors						Responses			
	X_1_	X_2_	X_3_	X_4_	X_5_	X_6_	Y_TP_ (mg·g^−1^)		Y_TA (_mg·g^−1^)	
							Experimental	Predicted	Experimental	Predicted
1	0	0	−1	0	−1	−1	55.4367	51.0831	20.7595	23.8541
2	0	0	1	0	−1	−1	48.6499	49.6621	29.8821	25.4294
3	0	0	−1	0	1	−1	51.4343	48.3304	29.0606	31.2985
4	0	0	1	0	1	−1	41.6667	42.334	27.9193	26.3493
5	0	0	−1	0	−1	1	46.1649	45.0747	27.6777	27.6226
6	0	0	1	0	−1	1	47.6107	51.1375	25.6501	25.0375
7	0	0	−1	0	1	1	50.4893	49.0542	28.4150	31.2426
8	0	0	1	0	1	1	45.7652	50.5417	23.6023	22.1329
9	0	−1	0	−1	−1	0	57.1168	57.3752	28.5256	24.7846
10	0	1	0	−1	−1	0	44.7735	48.3304	26.8602	29.2928
11	0	−1	0	1	−1	0	41.9739	53.0361	27.755	23.6113
12	0	1	0	1	−1	0	41.3703	46.5404	26.6112	23.4784
13	0	−1	0	−1	1	0	57.8351	55.2335	27.0548	34.781
14	0	1	0	−1	1	0	71.3186	57.688	27.1787	26.729
15	0	−1	0	1	1	0	41.3184	40.33	28.5542	30.715
16	0	1	0	1	1	0	48.1604	45.3335	18.8744	18.0219
17	−1	0	−1	−1	0	0	37.8811	41.5155	14.3354	19.5394
18	1	0	−1	−1	0	0	39.2436	41.1874	19.2532	42.8583
19	−1	0	1	−1	0	0	48.7908	48.0444	18.0553	17.6695
20	1	0	1	−1	0	0	43.5972	51.7521	21.1111	22.2834
21	−1	0	−1	1	0	0	54.6703	45.6802	17.6946	11.5099
22	1	0	−1	1	0	0	35.7746	37.3562	20.6987	26.097
23	−1	0	1	1	0	0	37.9611	35.1821	10.1680	24.5504
24	1	0	1	1	0	0	33.6931	30.8939	20.6241	20.4326
25	0	−1	−1	0	0	−1	39.6751	44.4043	25.5771	30.4887
26	0	1	−1	0	0	−1	42.5789	48.6886	26.0578	25.0639
27	0	−1	1	0	0	−1	43.7849	42.272	27.3046	29.0925
28	0	1	1	0	0	−1	42.0191	43.4037	24.5787	23.0862
29	0	−1	−1	0	0	1	47.4523	46.4907	27.6042	30.7219
30	0	1	−1	0	0	1	39.3819	41.3177	28.7060	28.5433
31	0	−1	1	0	0	1	58.3748	51.8422	25.7965	25.1653
32	0	1	1	0	0	1	48.6688	43.5167	28.9420	22.4052
33	−1	−1	0	0	−1	0	58.0268	57.5556	22.9897	19.5292
34	1	−1	0	0	−1	0	41.2425	32.9846	19.5373	27.3024
35	−1	1	0	0	−1	0	38.5507	36.2848	24.6490	32.9035
36	1	1	0	0	−1	0	46.8626	38.7149	20.2517	18.3034
37	−1	−1	0	0	1	0	35.7902	41.3694	17.7105	15.0653
38	1	−1	0	0	1	0	34.6257	34.3231	17.7143	48.8664
39	−1	1	0	0	1	0	20.7714	31.5978	19.0511	15.8794
40	1	1	0	0	1	0	48.5130	51.5526	19.2534	27.3073
41	−1	0	0	−1	0	−1	49.3362	46.1577	18.9945	14.4979
42	1	0	0	−1	0	−1	49.0123	49.4397	21.8380	28.2914
43	−1	0	0	1	0	−1	30.0802	28.3987	16.72389	13.4627
44	1	0	0	1	0	−1	24.1848	23.6849	20.7428	18.5244
45	−1	0	0	−1	0	1	35.7748	35.4395	16.4343	13.6402
46	1	0	0	−1	0	1	33.0203	35.537	19.5057	27.7794
47	−1	0	0	1	0	1	44.0929	44.5007	14.9678	13.5268
48	1	0	0	1	0	1	34.2591	36.6024	19.4501	18.9343
49	0	0	0	0	0	0	37.2240	45.8533	27.1667	23.3422
50	0	0	0	0	0	0	52.1910	45.8533	27.2028	23.3422
51	0	0	0	0	0	0	47.5538	45.8533	17.7051	23.3422
52	0	0	0	0	0	0	50.5994	45.8533	14.0658	23.3422
53	0	0	0	0	0	0	43.5576	45.8533	26.5943	23.3422
54	0	0	0	0	0	0	43.9939	45.8533	27.3184	23.3422
